# What Are Computer‐Assisted Methods Achieving in Fine‐Needle Aspiration Cytology of the Pancreas? A Systematic Review and Meta‐Analysis

**DOI:** 10.1111/cyt.70092

**Published:** 2026-05-26

**Authors:** Al‐Amaan Mohamed Mirzan, Roberto Dina

**Affiliations:** ^1^ Imperial College School of Medicine Imperial College London London UK; ^2^ Department of Metabolism, Digestion and Reproduction Imperial College London London UK

**Keywords:** computer‐assisted diagnosis, deep learning, diagnostic accuracy, image analysis, machine learning, meta‐analysis, pancreatic fine‐needle aspiration cytology, systematic review

## Abstract

**Objective:**

Pancreatic malignancies present major diagnostic challenges. The gold standard for diagnosis is endoscopic ultrasound‐guided fine‐needle aspiration (FNA) with cytopathological assessment. Workforce shortages have driven interest in computer‐assisted diagnosis (CAD) to improve efficiency. However, its overall diagnostic impact in pancreatic cytology remains unclear. We evaluated the accuracy of CAD in this setting.

**Methods:**

We conducted a systematic review and meta‐analysis. Five databases were searched (January 2010–May 2025) for studies applying CAD to pancreatic FNA cytology. Two reviewers independently screened studies, with risk of bias assessed using QUADAS‐2. Random‐effects bivariate models generated pooled sensitivity, specificity, and summary receiver‐operating‐characteristic (SROC) curves. Studies were analysed according to the unit of analysis: image‐level studies generated predictions from individual cytopathological images, whereas patient/case‐level studies integrated multiple images per case to produce a single diagnostic output.

**Results:**

Ten studies met eligibility criteria and provided quantitative data. At the image‐level, pooled sensitivity and specificity were 91% [95% confidence interval: 86–94] and 92% [87–96], respectively, with a SROC area under the curve (AUC) of 0.962. At the patient/case‐level, pooled sensitivity and specificity were 85% [69–93] and 91% [73–97], respectively, with an SROC AUC of 0.919. Heterogeneity was high (*I* ^2^ = 65%–93%), driven by retrospective designs and variable reference standards.

**Conclusion:**

CAD tools achieve near‐expert accuracy for definitive diagnoses, reducing routine screening burdens and accelerating surgical planning. However, selection bias, single‐centre training and inconsistent thresholds limit generalisability. Prospective multi‐centre validation using whole‐slide workflows is warranted, and telepathology integration could expand low‐resource access.

**Print Synopsis (For ‘Inside This Month's Cytopathology’):**

This systematic review and meta‐analysis shows that computer‐assisted diagnosis for pancreatic EUS‐FNA cytology achieves high accuracy in distinguishing benign from malignant lesions, with pooled sensitivity up to 91% and specificity up to 92%. Current evidence is dominated by retrospective and curated image‐level studies, highlighting the need for prospective multicentre validation using whole‐slide workflows before routine clinical adoption.

**Tweet‐Length Synopsis:**

Computer‐assisted diagnosis for pancreatic EUS‐FNA cytology shows promising accuracy in this systematic review and meta‐analysis, but current evidence is still limited by retrospective, curated datasets. Considerable prospective multicentre validation in real‐world whole‐slide workflows is needed before clinical adoption.

## Graphical abstract

This meta‐analysis demonstrates high diagnostic accuracy of computer‐assisted methods in pancreatic EUS‐FNA cytology (AUC 0.92–0.96) and supports an integrated, cytopathologist‐led workflow in which artificial intelligence functions as an adjunct to diagnostic interpretation and on‐site evaluation (ROSE). Created in BioRender. Mohamed Mirzan, A. (2026) https://BioRender.com/qnwwqfm.

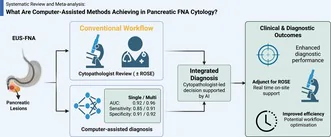

## 1 Introduction

Pancreatic malignancies remain among the deadliest solid tumours worldwide. GLOBOCAN 2020 estimated 496,000 new cases and 466,000 deaths, placing pancreatic cancer (PC) seventh for cancer‐related mortality despite its comparatively lower incidence [1]. Five‐year overall survival for all pancreatic neoplasms is only 1–12%, with minimal improvement over three decades [2]. Pancreatic ductal adenocarcinoma (PDAC) accounts for > 90% of cases, other types include cystic and endocrine tumours. Early treatment is critical: Stage IA cancers show 31.7% 5‐year survival versus < 1% in Stage IV [3]. However, vague symptoms such as abdominal discomfort, back pain, weight loss mean that fewer than 15% of patients present with resectable Stage I disease, while over 50% already have metastases at diagnosis [4]. An aging population and rising incidence could nearly double PC's absolute burden by 2040 [5]. Given the strong association between survival and stage at diagnosis, alongside the projected rise in disease burden, improving diagnostic accuracy and timeliness is of critical importance.

Increasing demand on diagnostic services, particularly cytopathology, has intensified the need for efficient and scalable tools. Following detection of a suspicious pancreatic lesion on CT or MRI, endoscopic ultrasound‐guided tissue acquisition, including EUS‐FNA, is a key component of pathological diagnosis [6]. EUS‐FNA is minimally invasive and achieves high sensitivity with few complications [7, 8]. However, diagnostic accuracy is limited by atypical cytological findings [9], tumour heterogeneity and a global shortage of cytopathologists [10, 11, 12, 13, 14]. These pressures are driving a shift to digital pathology, enabling remote interpretation and integration of tools such as digital image analysis (DIA) and artificial intelligence (AI).

While EUS‐FNA remains a cornerstone of pancreatic cancer diagnosis, its reliance on expert cytopathologist interpretation introduces variability that AI‐based systems are now positioned to address. AI and DIA systems learn cytological patterns to identify malignant features and highlight regions of interest for expert review, improving diagnostic speed, consistency and accessibility. This systematic review and meta‐analysis evaluated the current application of machine learning in pancreatic cytopathology, identified methodological gaps and proposed recommendations to accelerate clinical adoption of AI‐based diagnostic tools.

## 2 Methods

Our systematic review and meta‐analysis were conducted in line with PRISMA (Preferred Reporting Items for Systematic Reviews and Meta‐Analyses) guidelines and were registered under PROSPERO (ID: CRD420251014289). Our search was comprehensive and covered five databases: MEDLINE, EMBASE, Web of Science, Scopus and IEEE Xplore. The initial search strategy focused on AI applied to FNA cytology of PanNETs; however, preliminary screening showed that eligible studies were insufficient for robust meta‐analysis. Therefore, the scope was broadened to include all pancreatic lesions and computer‐assisted techniques, including DIA, WSI and other morphometric or machine‐learning methods. The original search was conducted up to 31‐03‐25, and a secondary search reflecting the revised inclusion criteria was carried out up to 07‐05‐25.

We used terminology based on computer‐assisted methods (including AI, ML, DL, DIA and neural networks), fine‐needle aspiration/biopsy cytology and morphology and pancreatic lesions (including pancreatic malignancy and benign conditions). There was no restriction on the specific computer‐assisted modality or type of cytology preparation (smear, cell block, fluid). Our full search strategy, designed to capture studies assessing the performance of these methods in detecting malignancy in pancreatic cytology, is outlined in Appendix A. We limited the search to English‐language human studies and used Covidence [15] to structure the screening process. Given the limited availability of studies, we did not restrict by report status and included conference abstracts.

### 2.1 Data Collection and Extraction

From each included study, we extracted key data. Extracted items included study design (retrospective or prospective), sample size and characteristics, types of pancreatic lesions evaluated (e.g., pancreatic ductal adenocarcinoma (PDAC), cystic neoplasms, PanNETs), details of the AI technique (algorithm type, features used, training method), reference standard for diagnosis (e.g., surgical histopathology or clinical follow‐up) and outcome measures (sensitivity, specificity, accuracy, area under the ROC curve). When not provided, 2 × 2 contingency data were derived from reported metrics. Discrepancies were resolved through discussion and source review. For Yucel et al. [16], grade‐specific cell counts and performance metrics were taken directly from their published tables.

Our primary outcome was overall diagnostic accuracy (the proportion correctly classified as benign vs. malignant). Secondary outcomes were sensitivity and specificity. When authors reported sensitivity/specificity without raw counts, we back‐calculated counts where possible.

### 2.2 Statistical Analysis

All analyses were completed in R [17] using R Studio [18] with the meta [19], mada [20], metafor [21], readxl [22], tidyverse [23] and caTools [24] packages. Diagnostic 2 × 2 tables (True Positive, False Positive, False Negative, True Negative) were extracted or backcalculated for every study; when any cell contained a zero, a uniform continuity correction of +0.5 was applied to all four cells to permit logit transformation. Studies were categorised based on the unit of analysis. Image‐level studies generated predictions from individual cytopathological images or fields of view. Patient/case‐level studies generated a single diagnostic output per case by integrating information from multiple images, fields or specimens. Sensitivity, specificity and overall accuracy were pooled using a PLOGIT generalised‐linear mixed model (metaprop), stratified by unit of analysis (image‐level vs. patient/case‐level). Each subgroup yielded inverse‐variance weighted proportions with 95% CI plus heterogeneity (*I* ^2^, *τ* ^2^); between‐group differences were tested with *χ* ^2^ (*Q*_*M*_, *p* < 0.05). A Reitsma bivariate random‐effects model (mada::reitsma) incorporated the unit‐of‐analysis moderator, and Wald tests assessed coefficient significance. Subgroup SROC curves (mada::sroc) with AUCs (mada::AUC, caTools::trapz) were plotted in ggplot2. Accuracy was pooled using the same framework. Sensitivity analyses excluded conference abstracts and removed continuity corrections.

## 3 Results

Our search identified 3701 records. After removal of 1306 duplicates, 2395 records were screened and 2302 were excluded at title and abstract level. 93 full texts were assessed for eligibility, of which 72 were excluded due to wrong study design, wrong setting or wrong intervention, leaving 21 studies included in the systematic review (Figure 1). These studies evaluated various computational methods, including artificial intelligence (AI) models, DIA and mathematical algorithms, applied to EUS‐FNA samples for tasks related to pancreatic pathology. Two reviewers independently identified relevant studies. Any discrepancies were resolved by author consensus.

**FIGURE 1:**
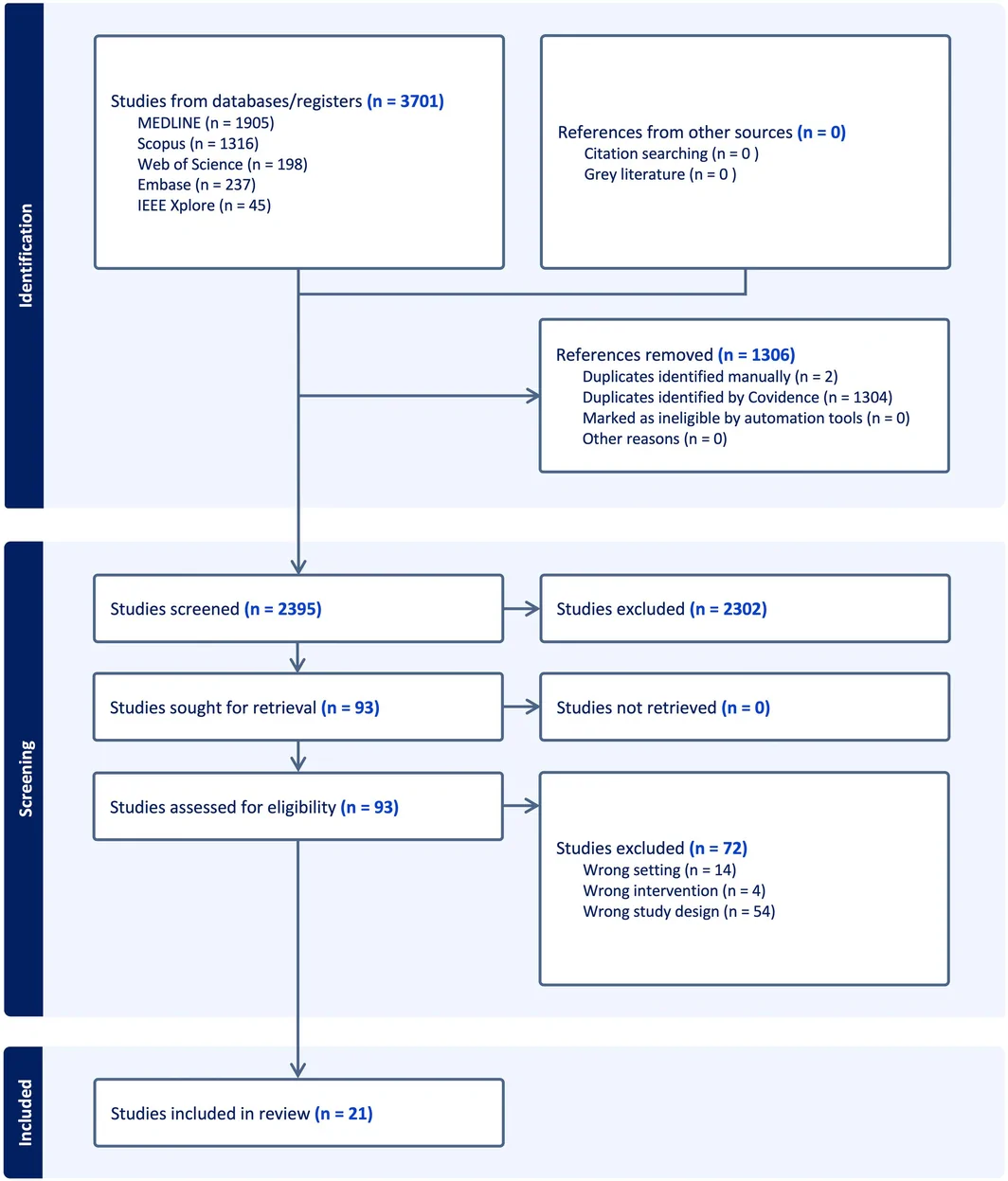
PRISMA flow diagram of study selection PRISMA flow diagram summarising the identification, screening, eligibility assessment and inclusion of studies in the systematic review. PRISMA, Preferred Reporting Items for Systematic Reviews and Meta‐Analyses.

Seventeen studies were retrospective cohorts, two were prospective clinical trials and two were technical feasibility papers. Most investigations originated from East Asia or North America, with additional single centre contributions from France and Iran. Sample sizes and units of analysis varied. Patient/case‐level cohorts ranged from 9 to 194 individuals, while image‐level datasets spanned 55 to more than 21,652 digitised cytology fields; the largest external test set contained 3213 images from 194 patients [25]. Reported mean ages clustered in the late 50s to early 60s, and the male‐to‐female ratio was roughly balanced across studies. All studies analysed material obtained by EUS‐FNA and fine‐needle biopsy cell blocks.

Key descriptive variables are summarised in Table 1, with model characteristics and tasks in Table 2. Tables 3 and 4 detail cohort definitions, diagnostic classifications and preparation methods and Table 5 summarises image acquisition and analytical approaches for the studies included in the meta‐analysis.

**TABLE 1:** Study characteristics and patient demographics of included studies.

Author	Year	Type of study	Country	Outcome	Time period	Gender (M)	Age (mean ± SD)
Azzouz et al. [26]	2021	Retrospective diagnostic accuracy	France	Accuracy	2017–2020	NR	NR
Fujii et al. [27]	2024	Retrospective diagnostic accuracy	Japan	Sensitivity, specificity, accuracy, PPV, NPV, AUC	Apr 19–Sep 22	58.0	71.24 ± 7.4
Hashimoto et al. [28]	2018	Retrospective diagnostic accuracy	Japan	Sensitivity, specificity, accuracy	2015–2015	NR	NR
Hashimoto et al. [29]	2020	Prospective diagnostic accuracy	Japan	Sensitivity, specificity, accuracy	2015–2017	NR	NR
Khozeymeh et al. [30]	2023	Retrospective cross‐sectional	Iran	Sensitivity, specificity, cutoff points, accuracy	2018–2019	55.6	59.3 ± 15.8
Kong et al. [31]	2019	Prospective clinical trial	China	Sensitivity, specificity, accuracy	Oct 14–Oct 16	66.0	71.3 ± 7.4
Lin et al. [32]	2023	Retrospective diagnostic accuracy	China	Sensitivity, specificity, accuracy, PPV, NPV	Jan 21–Jan 22	58.8	58.73 ± 11.57
Momeni‐Boroujeni et al. [33]	2017	Retrospective diagnostic accuracy	USA	Sensitivity, specificity, accuracy	Jan 10–Jul 17	NR	NR
Qin et al. [34]	2023	Retrospective diagnostic accuracy	China	Sensitivity, specificity, accuracy, AUC	Jan 20–Mar 23	65.3	60.2 ± 13.2
Sohn et al. [35]	2023	Retrospective diagnostic accuracy	USA	Balanced accuracy, weighted *F*1‐score, weighted AUROC and AUPRC	NR	54.0	69.24
Yamda et al. [36]	2022	Exploratory observational	Japan	Sensitivity, specificity, accuracy	NR	NR	NR
Zhang et al. [37]	2023	Retrospective diagnostic accuracy	China	Sensitivity, specificity, accuracy, PPV, NPV	2019–2021	NR	NR
Zhang et al. [25]	2022	Retrospective multicentre diagnostic	China	Sensitivity, specificity, accuracy	Jan 16–Jan 19	59.8	62.1 ± 11.6
Zhang et al. [38]	2024	Retrospective diagnostic accuracy	China	Sensitivity, accuracy	2019–2022	NR	NR
Madrigal et al. [39]	2021	Retrospective feasibility	USA	Feasibility study	2011–2018	NR	NR
Shaker et al. [40]	2024	Retrospective diagnostic accuracy	USA	Accuracy	Jan 19–Dec 22	57.6	62.3
Ishikawa et al. [41]	2022	Retrospective diagnostic accuracy	Japan	Sensitivity, specificity, accuracy, PPV, NPV, AUC	Sep 19–Jan 21	61.5	67.58 ± 10.93
Ishikawa et al. [41]	2022	Retrospective diagnostic accuracy	Japan	Sensitivity, specificity, accuracy, PPV, NPV, AUC	Sep 19–Jan 21	66.7	65 ± 10.37
Fang et al. [42]	2022	Retrospective diagnostic accuracy	China	Sensitivity, specificity, accuracy, PPV, NPV	Jan 18–Apr 24	61.0	61.64 ± 11.95
Thosani et al. [43]	2021	Retrospective diagnostic validation	USA	Sensitivity, specificity, accuracy	NR	NR	NR
Thosani et al. [44]	2024	Prospective clinical trial	USA	Adequacy, accuracy	Oct 21–May 22	NR	NR
Patel et al. [45]	2024	Retrospective diagnostic validation	USA	Accuracy, interobserver variability	NR	NR	NR

*Note:* Overview of each study's design, country, diagnostic outcomes, sample collection period and basic patient demographics (gender and age). Age was estimated assuming normal distribution in some papers as full data was unavailable (Fujii et al. [27], Kong et al. [31]) and for other studies we used averages of all data given to simplify it into a single figure. Time period refers to when the samples were originally obtained.

Abbreviations: AUC, area under the curve; AUPRC, area under the precision‐recall curve; AUROC, area under the receiver operating characteristic curve; NPV, negative predictive value; PPV, positive predictive value.

**TABLE 2:** Model characteristics and diagnostic performance of all studies included in this systematic review.

Author	Model	Task	Sensitivity	Specificity	Accuracy	AUC
Azoouz et al. [26]	VGG‐19, ResNet‐50 and DenseNet‐201	Malignant vs. Benign	NR	NR	0.97	NR
Fujii et al. [27]	ROSE‐AI model	Malignant vs. Benign	0.91	0.85	0.88	0.95
Hashimoto et al. [28]	Deep learning	Malignant vs. Benign	0.80	0.80	0.80	NR
Hashimoto et al. [29]	ImageNet CNNbn	Malignant vs. Benign	0.96	0.90	0.93	NR
Khozeymeh et al. [30]	Digital image analysis	Malignant vs. Benign	0.60	0.87	0.72	0.80
Kong et al. [31]	Digital image analysis	Malignant vs. Benign	0.84	0.80	0.83	NR
Lin et al. [32]	ROSE‐AI model	Malignant vs. Benign	0.78	0.91	0.89	NR
Momeni‐Boroujeni et al. [33]	Multilayer perceptron neural network	Malignant vs. Benign	0.80	0.75	0.77	NR
Qin et al. [34]	ResNet‐18 with SimSiam	Malignant vs. Benign	0.93	0.91	0.92	NR
Sohn et al. [35]	MIPCL (deep learning framework)	Malignant vs. Benign	NR	NR	0.91	0.94
Yamda et al. [36]	Mathematical technology for cytopathology for ROSE	Malignant vs. Benign	0.78	0.70	0.75	NR
Zhang et al. [37]	MSHT	Malignant vs. Benign	0.93	0.97	0.96	NR
Zhang et al. [25]	DCNN	Malignant vs. Benign	0.94	0.92	0.94	0.96
Zhang et al. [38]	SI‐ViT	Malignant vs. Benign	0.91	NR	0.94	NR
Madrigal et al. [39]	AI + Digital image analysis	Grading PanNET	NR	NR	NR	NR
Shaker et al. [40]	Digital image analysis	Grading PanNET	NR	NR	NR	NR
Ishikawa et al. [41]	Contrastive learning SimCLR	Evaluate EUS‐FNB	0.90	0.54	0.84	NR
Ishikawa et al. [41]	Deep learning—AlexNet	Evaluate EUS‐FNB	0.86	0.55	0.72	NR
Fang et al. [42]	SCNN system	Diagnose	0.94	0.95	0.94	0.99
Thosani et al. [43]	ROSE‐AId	Assess adequacy	0.92	0.44	0.82	NR
Thosani et al. [44]	ROSE‐AId	Assess adequacy	1.00	0.40	0.92	NR
Patel et al. [45]	ROSE‐AId	AI vs. Physicians	NR	NR	0.87	NR

*Note:* Summary of each study's AI model, diagnostic task and reported performance metrics (sensitivity, specificity, accuracy and AUC).

Abbreviations: AlexNet, convolutional neural network architecture; AUC, area under the curve; CNNbn, convolutional neural network with batch normalisation; DCNN, deep convolutional neural network; DenseNet‐201, densely connected convolutional network (201 layers); MIPCL, multiple instance probability contrastive learning; MSHT, multi‐scale hierarchical transformer; ResNet‐18, residual neural network (18 layers); ResNet‐50, residual neural network (50 layers); ROSE‐AI, rapid on‐site evaluation—artificial intelligence; SCNN, sequential convolutional neural network; SimCLR, simple framework for contrastive learning of visual representations; SI‐ViT, self‐supervised vision transformer; VGG‐19, visual geometry group network (19 layers).

**TABLE 3:** Cohort characteristics and diagnostic classification of included studies.

Study	Cohort size (patients/images)	Diagnostic categories
Azzouz et al. [26]	72 patients/21,652 tile images	Tumoral vs. non‐tumoral
Fujii et al. [27]	45 patients/4159 divided images	Adenocarcinoma (36 cases) vs. non‐malignancy (9 cases)
Hashimoto et al. [28]	23 cases/450 images	Suggestive/suspicious of malignancy vs. suggestive of benign
Hashimoto et al. [29]	1840 specimens (1440 training, 400 validation)	PDAC vs. Benign (defined as non‐diagnostic or non‐malignant)
Khozeymeh et al. [30]	115 patients/150 digital tumoral cell images	6‐tier system: Non‐diagnostic (4), negative (27), atypical (7), neoplastic (16), suspicious (3), positive (58)
Kong et al. [31]	142 patients (87 excluded for inadequacy)	4‐tier: Benign, atypical, suspicious, malignant (87 non‐diagnostic cases excluded)
Lin et al. [32]	51 patients/1160 images	6‐tier: Non‐diagnostic, negative, atypical, neoplastic, suspicious, positive/malignant
Momeni‐Boroujeni et al. [33]	75 cases/277 filtered images	Negative (24), atypical (31), suspicious/positive (20)
Qin et al. [34]	72 patients/1913 pairs of RGB & hyperspectral images	Negative (38.9%), atypical (12.5%), suspicious (8.3%), positive (40.3%)
Sohn et al. [35]	102 cases/2000 regional WSIs	Strictly evaluated as regions positive vs. negative for adenocarcinoma
Yamda et al. [36]	246 specimens	Normal (103 specimens) vs. adenocarcinoma (143 specimens)
Zhang et al. [37]	4240 sampled images	Cancer (1518 images) vs. normal pancreatic cells (2722 images)
Zhang et al. [25]	194 patients/5345 images	Cancer (113), mild atypia (23), other tumours (11), no tumours (47)
Zhang et al. [38]	5088 images	Pancreatic cancer (1773) vs. normal (3315)
Madrigal et al. [39]	17 PanNET cases	Categorised by Ki‐67 proliferative index: WHO Grades 1 (*n* = 5), 2 (*n* = 1) and 3 (*n* = 1)
Shaker et al. [40]	66 consecutive cytologic specimens	WHO Grades: G1 (< 3%), G2 (3%–20%), G3 (> 20%)
Ishikawa et al. [41]	63 patients/98 specimens (evaluated via 298 images)	Evaluated for macroscopic adequacy: Histologically diagnosable (81) vs. undiagnosable (17)
Ishikawa et al. [41]	96 patients/173 specimens	Histologically diagnosable (145) vs. undiagnosable (28)
Fang et al. [42]	210 patients/3213 images	PDAC (100 patients), non‐PDAC (110). Separately collected 33 explicit ‘Atypical’ images
Thosani et al. [43]	> 400 cases for training/77 unique validation images	Assessed for adequacy (adequate vs. inadequate) and diagnosis (61 Malignant vs. 16 Benign)
Thosani et al. [44]	50 patients enrolled (39 analysed in final cohort)	Adenocarcinoma (32 cases), NET (2 cases), Benign (5 cases)
Patel et al. [45]	77 unique cytopathology images	Malignant PSLs (61 images) vs. Benign tissue representations (16 images)

*Note:* Summary of included studies reporting publication year, cohort size (patients, cases, specimens or images) and diagnostic categories used for model evaluation. Cohort sizes are presented as reported by the original studies and may include training and validation datasets where specified. Diagnostic categories are shown as defined within each study.

Abbreviations: NET, neuroendocrine tumour; PanNET, pancreatic neuroendocrine tumour; PDAC, pancreatic ductal adenocarcinoma; WSI, whole‐slide image.

**TABLE 4:** Tumour types, malignancy classification and cytology preparation of included studies.

Study	Tumour *t*ypes	Malignancy *c*lassification	Cytology preparation
Azzouz et al. [26]	PDAC	Pancreatic adenocarcinoma	FNA cytopunctures; HES‐stained and digitised into WSIs
Fujii et al. [27]	PDAC, non‐malignancy (autoimmune pancreatitis, chronic pancreatitis)	PDAC vs. autoimmune & chronic pancreatitis	EUS‐FNA direct smears; Diff‐Quik stain
Hashimoto et al. [28]	PDAC	Pancreatic cystic lesions (IPMN, SCA, NET, Pseudocyst)	EUS‐FNA cytology smears & needle‐based confocal laser endomicroscopy (nCLE)
Hashimoto et al. [29]	PDAC, non‐malignancy	Pancreatic ductal adenocarcinoma	EUS‐FNA cytology specimens for Rapid On‐Site Evaluation (ROSE)
Khozeymeh et al. [30]	Pancreatic lesions	Broadly categorised as ‘pancreatic malignancy’	EUS‐FNA cytological smears; H&E stain
Kong et al. [31]	PDAC, squamous cell, PANNET, acinar cell	Mixed: Adenocarcinoma, squamous cell, NET, acinar cell	EUS‐FNA Liquid‐based cytology (ThinPrep); Feulgen staining
Lin et al. [32]	PDAC (94.1%) or other celiac lesions (5.9%)	Solid pancreatic masses (94.1%) & other celiac lesions	EUS‐FNA air‐dried direct smears; Diff‐Quik (D&F) stain
Momeni‐Boroujeni et al. [33]	Benign, malignant adenocarcinoma and atypical cancers.	Exocrine carcinoma (ductal adenocarcinoma)	FNA biopsy cell clusters on ThinPrep liquid‐based slides; Pap stain
Qin et al. [34]	PDAC, benign pancreatic cells (pseudo tumoral chronic pancreatitis, autoimmune pancreatitis)	PDAC vs. Pseudotumoral chronic & autoimmune pancreatitis	EUS‐FNA Liquid‐based cytology (LBC); H&E stain
Sohn et al. [35]	PDAC, chronic pancreatitis, mucinous cystic neoplasm, IPMN, undifferentiated carcinoma, metastatic renal cell carcinoma, pancreatic pseudocyst	Mixed: PDAC, chronic pancreatitis, MCN, IPMN, undifferentiated carcinoma, metastatic RCC	EUS‐FNA Diff‐Quik and Papanicolaou (Pap) smear preparations
Yamda et al. [36]	PDAC, non‐malignancy	Adenocarcinoma	Direct smears (Diff‐Quik) for ROSE & formalin‐fixed core biopsy specimens via EUS‐FNA/FNB
Zhang et al. [37]	Pancreatic lesions	Pancreatic cancer	EUS‐FNA Diff‐Quik Giemsa stained smears during ROSE examinations
Zhang et al. [25]	PDAC, mild atypical lesions, PanNET, solid pseudopapillary tumours, chronic pancreatitis, autoimmune pancreatitis	Adenocarcinoma vs. PanNET, SPPT and non‐tumours (chronic/autoimmune pancreatitis)	EUS‐FNA cytopathological slides; H&E or Liu's (modified Romanowsky) stain
Zhang et al. [38]	PDAC, non‐malignancy	Pancreatic cancer	EUS‐FNA liquid biopsy samples; Diff‐Quik Giemsa during ROSE
Madrigal et al. [39]	PDAC	Pancreatic neuroendocrine tumours (PanNET)	FNAB Formalin‐fixed paraffin‐embedded (FFPE) cell blocks; Ki‐67 IHC
Shaker et al. [40]	PanNET, Pancreatic small cell carcinoma	Pancreatic neuroendocrine neoplasms (PanNEN) and NECs	FFPE cell block sections; Ki‐67 IHC scanned into WSIs
Ishikawa et al. [41]	PDAC, mass‐forming pancreatitis, autoimmune pancreatitis, PanNET, pancreatic metastasis, intraductal papillary mucinous carcinoma	Mixed: PDAC, mass‐forming pancreatitis, AIP, PanNET, metastasis, IPMC	Stereomicroscopic images of EUS‐FNB macroscopic core tissue (MOSE)
Ishikawa et al. [41]	PDAC, mass‐forming pancreatitis, autoimmune pancreatitis, PanNET, pancreatic metastasis, intraductal papillary mucinous carcinoma	Mixed: PDAC (66), mass‐forming pancreatitis (13), AIP (11), PanNET (3), etc.	EUS‐FNB stereomicroscopic images (MOSE) explicitly mapped against H&E sections
Fang et al. [42]	Neuroendocrine tumours, including PanNET	PDAC vs. non‐PDAC (chronic pancreatitis, autoimmune pancreatitis, PanNET)	EUS‐FNA cytological smears (excluded cases with > 50% erythrocyte contamination)
Thosani et al. [43]	Not reported	PDAC (39), NET (22) vs. Benign aetiologies (acinar, ductal, inflammatory, RBCs)	EUS‐FNA cytology slides evaluated using ROSE‐AId software
Thosani et al. [44]	Not reported	Adenocarcinoma, NET vs. Granulomatous & chronic pancreatitis	EUS‐FNA samples evaluated directly on‐site (ROSE‐AId)
Patel et al. [45]	Not reported	Malignant PSLs (PDAC, NET) vs. Benign (acinar, ductal, GI epi, inflammatory, RBCs)	Cytopathology images derived from EUS‐FNA slides

*Note:* Summary of tumour types included, diagnostic classification frameworks and cytology preparation methods used across studies. Tumour types and classification categories are reported as defined by the original studies. Cytology preparation includes specimen type, staining methods and slide preparation techniques.

Abbreviations: AIP, autoimmune pancreatitis; EUS‐FNA, endoscopic ultrasound fine‐needle aspiration cytology; FFPE, formalin‐fixed paraffin‐embedded; FNA, fine‐needle aspiration; FNB, fine‐needle biopsy; H&E, haematoxylin and eosin; IHC, immunohistochemistry; IPMN, intraductal papillary mucinous neoplasm; LBC, liquid‐based cytology; MCN, mucinous cystic neoplasm; NET, neuroendocrine tumour; PanNEN, pancreatic neuroendocrine neoplasm; PanNET, pancreatic neuroendocrine tumour; PDAC, pancreatic ductal adenocarcinoma; RCC, renal cell carcinoma; ROSE, rapid on‐site evaluation; SCA, serous cystadenoma; SPPT, solid pseudopapillary tumour; WSI, whole‐slide image.

**TABLE 5:** Image acquisition methods and analytical approach of studies included in the meta‐analysis.

Study	Image acquisition method	WSI	Field selection	Analysis level	Notes
Fang et al. (2025) [42]	Olympus BX53F2 microscope connected to a digital pathology reporting system (Path QC)	Non‐WSI	Manual: images were reviewed by a senior cytopathologist and inferior quality images were eliminated	Image‐level	Evaluated at a unified 400× magnification; explicitly excluded smears with > 50% erythrocyte contamination
Fujii et al. (2024) [27]	VENTANA iScan HT virtual slide scanner	WSI	Automated: WSIs were divided into 256 × 256 pixel images and tiles exceeding a cell‐area threshold were automatically extracted	Image‐level	The divided WSI images were pre‐processed for stain colour normalisation to maintain consistency
Lin et al. (2023) [32]	Olympus BX53F or IX73 microscope camera	Non‐WSI	Manual: Representative images were obtained, partially selected and then classified by a pathologist	Image‐level	Images were captured at 200× magnification and tiled into 256 × 256 pixel sizes
Qin et al. (2023) [34]	Olympus DP73 camera scanner for RGB; homemade medical hyperspectral imaging (MHSI) system	Non‐WSI	Manual: Pathologists classified cases and manually delineated the Regions of Interest (ROIs) on the images	Image‐level	1913 matched pairs of RGB and Hyperspectral (HSI) images were analysed to compare diagnostic accuracy
Zhang et al. (2023—MSHT) [37]	Basler ScA1 and Olympus DP73 digital cameras mounted on Olympus BX53 and Nikon Eclipse Ci‐S microscopes	Non‐WSI	Manual: images were independently sampled under the supervision of senior pathologists	Image‐level	All images were resized to 1390 × 1038 to maintain a uniform 400× magnification factor
Kong et al. (2019) [31]	Landing Medical High‐Tech automated image analyser	Non‐WSI	Automated: the system automatically loaded each slide, scanned the area of ThinPrep cell deposition and collected images of every detected object	Patient/Case‐level	87 patients were excluded due to inadequacy of cellular yield for digital analysis
Momeni‐boroujeni et al. (2017) [33]	Olympus DP73 camera mounted on an Olympus BX40 microscope	Non‐WSI	Manual: high‐resolution images were filtered by a cytopathologist so only highly representative cell clusters were included	Patient/Case‐level	Used K‐means clustering to segment images into regions of interest; captured at 400× original magnification
Zhang et al. (2022—DCNN) [25]	Olympus DP73 camera mounted on an Olympus BX40 microscope	Non‐WSI	Manual: an experienced certificated cytopathologist delineated along the margin of the cell clusters and labelled regions at the pixel level	Patient/Case‐level	The algorithm evaluated slide adequacy and required three positive images per patient to render a malignant diagnosis
Khozeymeh et al. (2023) [30]	Olympus CX31 microscope with a 12‐megapixel digital camera	Non‐WSI	Manual: selecting specific clusters where cell and nucleus details were clear and did not overlap	Patient/Case‐level	Pictures were taken at 10×, 20× and 40×, with the 400× magnification ultimately selected for analysis.
Yamada et al. (2022) [36]	Not explicitly reported. Evaluated via mathematical image processing on static specimens	Non‐WSI	Manual: the cytologist manually selected the region of interest to remove unnecessary overlap of cell clusters	Patient/Case‐level	Utilised univariate and multivariate mathematical analysis (reaction–diffusion method) to classify normal vs. adenocarcinoma tissues

*Note:* Summary of image acquisition methods, use of whole‐slide imaging (WSI), field selection approach and level of analysis for studies included in the meta‐analysis. Field selection refers to whether regions of interest were selected manually or automatically. Analysis level indicates whether models generated predictions at the image‐level or patient/case‐level.

Abbreviations: HSI, hyperspectral imaging; RGB, red‐green‐blue imaging; ROI, region of interest; WSI, whole‐slide image.

Of the 21 studies included in the systematic review, 10 provided sufficient quantitative data for differentiating malignant (primarily PDAC) versus benign solid pancreatic lesions using computational analysis on EUS‐FNA derived cytology samples. These were included in the meta‐analysis and stratified by unit of analysis into patient/case‐level (*n* = 5 datasets: Khozeymeh et al. 2023 [30], Kong et al. 2019 [31], Momeni‐Boroujeni et al. 2017 [33], Yamada et al. 2022 [36], Zhang et al. 2022 [25]) and image‐level (*n* = 5 datasets: Fang et al. 2025 [42], Lin et al. 2023 [32], Qin et al. 2023 [34], Fuji et al. 2024 [27], Zhang et al. 2023 [37]). The full true‐positive, false‐positive, false‐negative and true‐negative dataset is available in Table S1.

Upon Quality Assessment of Diagnostic Accuracy Studies 2 (QUADAS‐2) assessment, applicability concerns were high or unclear for patient selection and the index test in most studies. Although the overall clinical question aligned, AI workflows deviated from routine practice, with most models analysing curated, human‐selected ‘hot spots’ rather than autonomous whole‐slide images. Patient selection was similarly affected by dataset simplification, as several studies excluded diagnostically challenging or blood‐obscured cases.

Risk of bias varied across studies. Patient selection (D1) was the most frequent source of bias, with nine studies rated high risk due to retrospective or case‐control designs without consecutive enrolment. The index test (D2) showed high or unclear risk in three studies due to post‐hoc threshold optimisation or exploratory modelling. The reference standard (D3) was low risk in eight studies using histology or clinical follow‐up, but high risk in two relying on cytology alone. Flow and timing (D4) was high risk in four studies due to post‐hoc exclusion of non‐diagnostic or low‐quality samples. These findings are summarised in the QUADAS‐2 traffic light plot (Figure 2).

**FIGURE 2:**
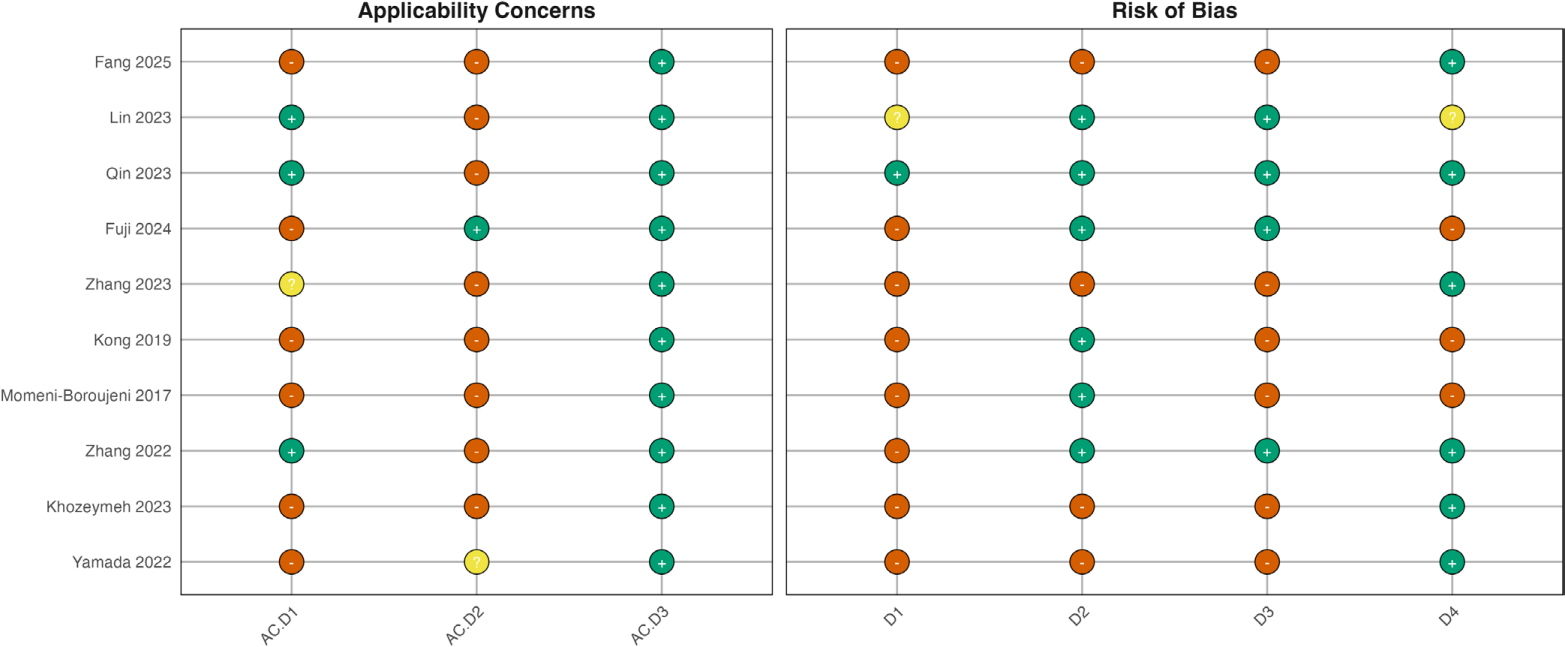
QUADAS‐2 assessment of risk of bias and applicability concerns for studies included in meta‐analysis Each domain is represented by a coloured symbol indicating the level of concern or risk: Green for low, yellow for unclear and orange for high. Applicability concerns are shown in the left panel and risk of bias in the right panel. Domains: Risk of bias—D1: Patient Selection; D2: Index Test; D3: Reference Standard; D4: Flow and Timing. Applicability concerns—AC.D1: Patient Selection; AC.D2: Index Test; AC.D3: Reference Standard.

### 3.1 Sensitivity Analysis

Patient/case‐level studies achieved a pooled sensitivity of 0.85 (95% CI 0.69–0.93), whereas image‐level studies achieved 0.91 (95% CI 0.86–0.94); the subgroup difference was not statistically significant (*χ* ^2^ = 1.20, *p* = 0.27). Substantial heterogeneity was present in both subgroups (*I* ^2^ = 80.4% and 77.6%, respectively).

### 3.2 Specificity Analysis

Patient/case‐level studies achieved a pooled specificity of 0.91 (95% CI 0.73–0.97), whereas image‐level studies achieved 0.92 (95% CI 0.86–0.96); the subgroup difference was not statistically significant (*χ* ^2^ = 0.09, *p* = 0.76). Heterogeneity was substantial, particularly in the image‐level subgroup (*I* ^2^ = 65.2% and 90.0%, respectively).

### 3.3 Accuracy

Reported study‐level accuracy ranged from 0.71 to 0.96 (Table 2). Image‐level algorithms demonstrated a higher median accuracy (≈0.90) compared to patient/case‐level models (≈0.82), although this difference was not clearly distinct across studies. Given that accuracy is influenced by the malignant‐to‐benign ratio within each dataset, pooled estimates were not calculated. Instead, sensitivity and specificity (Figures 3 and 4), which are independent of disease prevalence, were used for cross‐study comparison. overall diagnostic performance was further assessed using summary receiver operating characteristic (SROC) curves.

**FIGURE 3:**
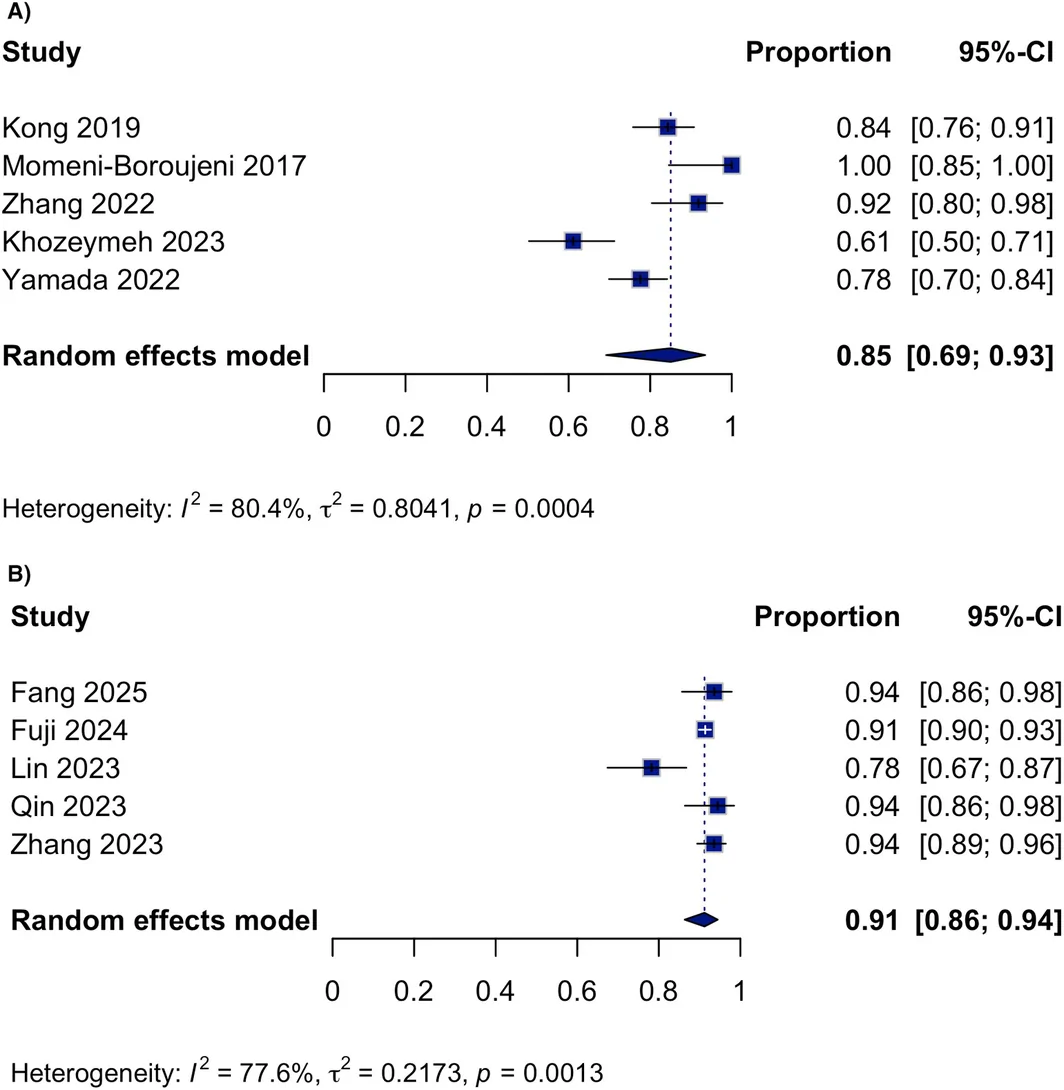
Computer assisted models demonstrate high pooled sensitivity for classifying pancreatic fine needle aspiration cytology, with no significant difference between patient/case‐level versus image‐level analyses Forest plot showing studyspecific and pooled sensitivities for computer aided diagnosis of solid‐ pancreatic lesions on fine‐needleaspiration (FNA) cytology. Forest plot showing study‐specific and pooled sensitivities for computer‐aided diagnosis of solid pancreatic lesions on fine‐needle aspiration (FNA) cytology. Upper panel represents patient/case‐level studies (A) versus image‐level studies (B). Squares represent individual study point estimates and horizontal bars indicating 95% confidence intervals (CI). Diamonds denote subgroup and overall random‐effects summary estimates; their width reflects the corresponding 95% CI. Betweenstudy heterogeneity is quantified by *I* ^2^ and *τ* ^2^ for each subgroup and overall. The dashed vertical line marks the overall pooled sensitivity.

**FIGURE 4:**
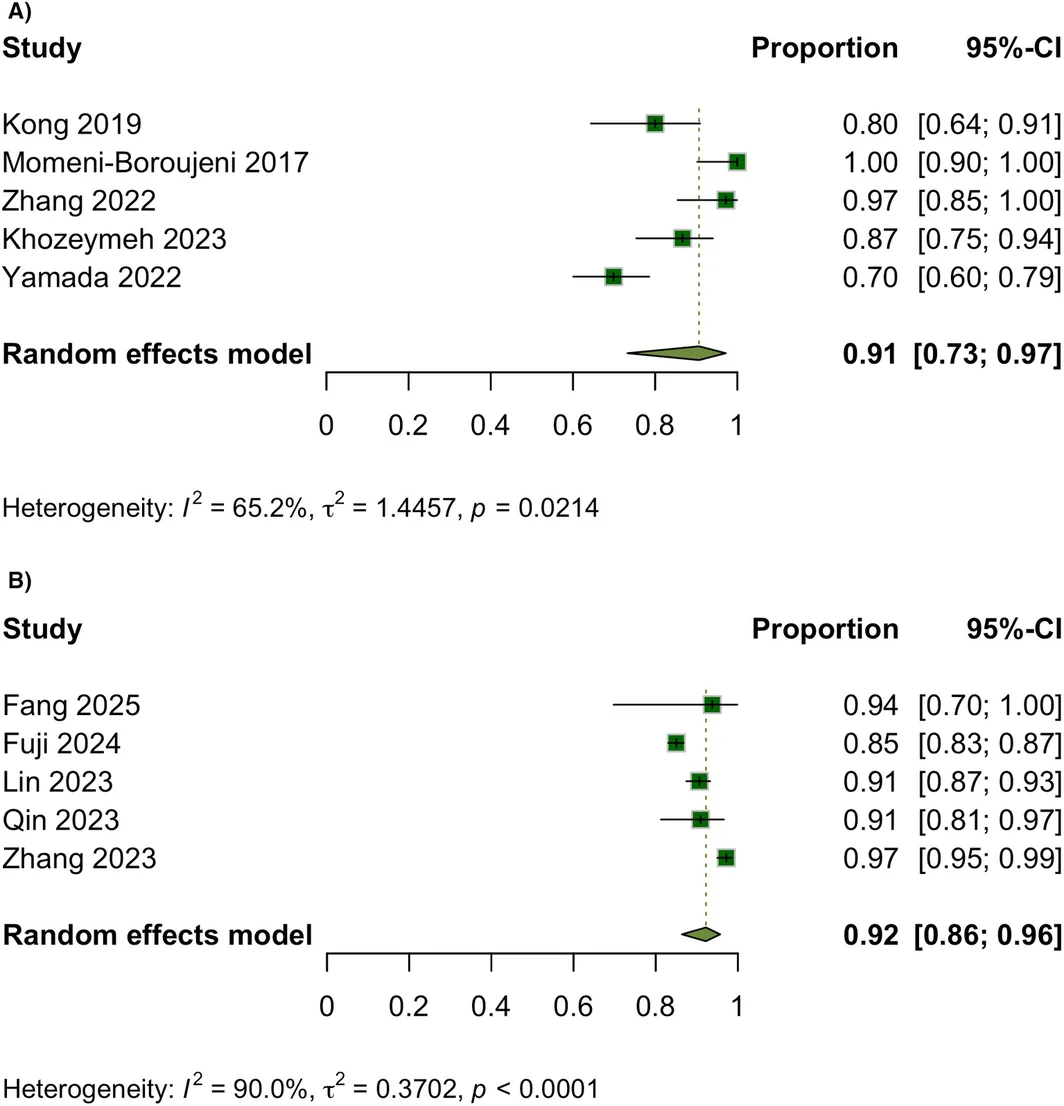
Computer‐aided systems achieve a high pooled specificity for classifying pancreatic FNA cytology, with no significant difference between patient/case‐level vs image‐level analyses. Specificities of individual studies (squares) and random‐effects summary estimates (diamonds) computer‐aided diagnosis of solid pancreatic lesions on fine‐needle aspiration (FNA) cytology. Results are stratified by the unit of analysis: Patient/case‐level datasets (A) versus image‐level datasets (B). Square size is proportional to inverse variance weight; horizontal bars show 95% confidence intervals (CI). Diamonds depict pooled specificity, with their width representing the 95% CI. The dotted vertical line marks the overall summary estimate. Statistical heterogeneity within each stratum and overall is expressed as *I* ^2^ (percentage of total variation attributable to between‐study differences) and *τ* ^2^ (between‐study variance). A *χ* ^2^ test evaluates whether pooled specificities differ between strata (*p* = 0.76).

### 3.4 SROC Curve

Two SROC curves were generated, stratified by unit of analysis: Patient/case‐level (Figure 5A) and image‐level (Figure 5B). The area AUC was 0.919 for patient/case‐level models and 0.962 for image‐level models. Individual study estimates are shown in Figure 5.

**FIGURE 5:**
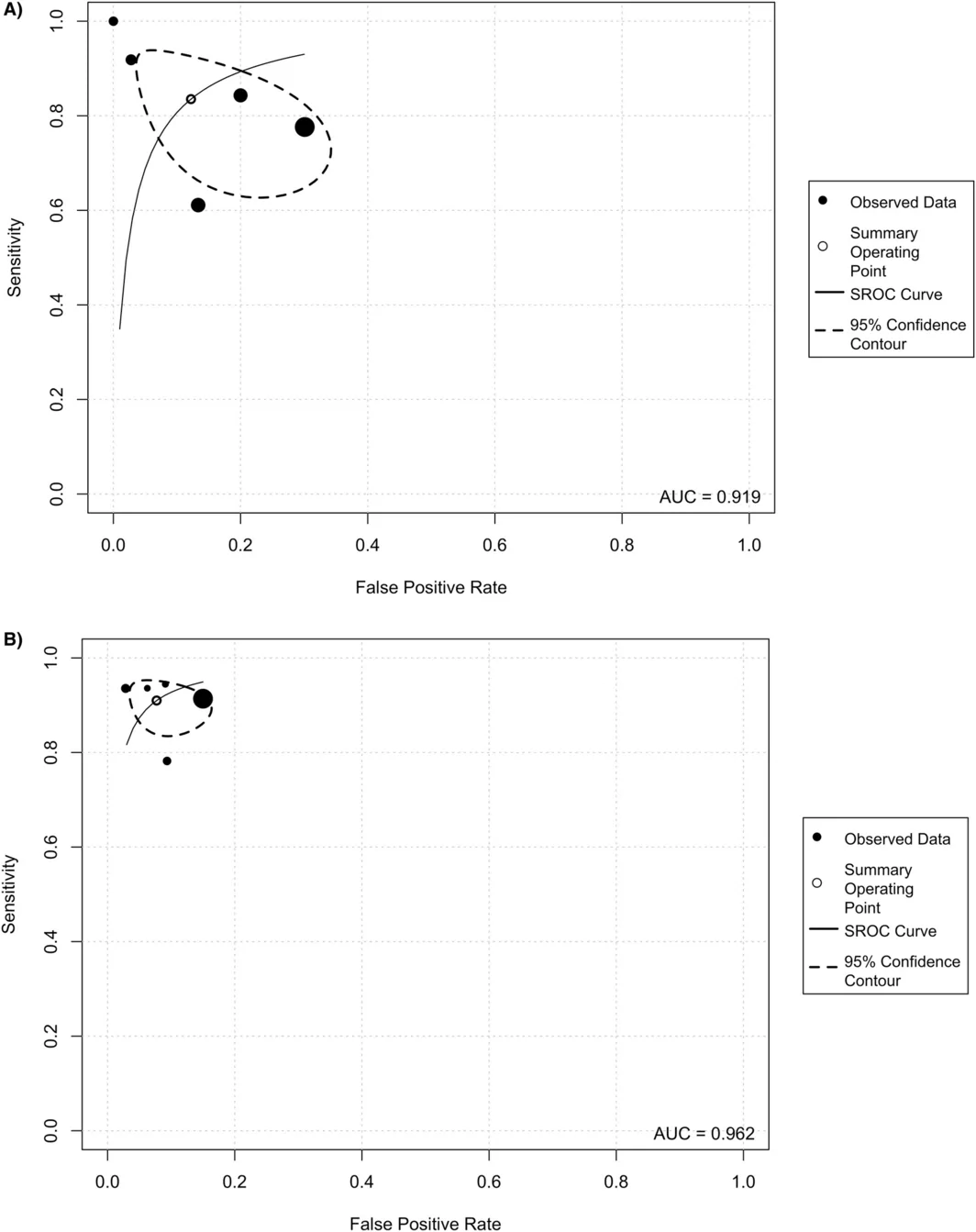
Summary receiver operating characteristic (SROC) curve for diagnostic performance by unit of analysis. The SROC curves summarise the sensitivity and specificity of included studies stratified by unit of analysis. Each point represents a study, with point size proportional to the study's sample size. The empty circle denotes the summary operating point (mean sensitivity and specificity) and the dashed line indicates the 95% confidence contour. The solid curve represents the modelled summary performance across studies. The upper panel (A) corresponds to patient/case‐level analyses, and the lower panel (B) to image‐level analyses. AUC, area under the curve.

The remaining studies could not be incorporated into a single meta‐analysis because they addressed highly variable tasks, employed different targets and used bespoke performance metrics. Some focused on automated grading (e.g., Ki‐67 hotspot quantification versus dual‐path CNN cytology grading), others on slide‐adequacy triage (flagging inadequate FNA smears) or AI‐assisted rapid on‐site evaluation (ROSE) with unique sensitivity, specificity and accuracy thresholds. Given this diversity in clinical questions and outcomes, a pooled effect estimate would be misleading. Instead, we present each study narratively to illustrate the range of AI approaches explored in pancreatic cytopathology.

## 4 Discussion

Our findings indicate that computer‐assisted diagnostic (CAD) methods achieve high accuracy in distinguishing benign from malignant pancreatic FNA cytology. Both patient/case‐level and image‐level analyses demonstrated strong diagnostic capability, with pooled sensitivities of 0.85 and 0.91 and specificities of 0.91 and 0.92, respectively. Importantly, subgroup analyses revealed no statistically significant differences in diagnostic performance between the image‐level and patient/case‐level models for either sensitivity (*χ* ^2^ = 1.20, *p* = 0.27) or specificity (*χ* ^2^ = 0.09, *p* = 0.76). The SROC curves supported this, with AUC values of 0.919 and 0.962, indicating strong threshold‐independent discrimination, particularly among image‐level models. However, these findings should not be taken as evidence that CAD can replace expert cytopathologists. The true value of the cytopathologist lies not in binary classification alone, but in integrating morphologic findings with clinical and radiologic context, assessing specimen adequacy and navigating diagnostic uncertainty. That multimodal interpretive role remains beyond the reach of current algorithms.

Our review nevertheless suggests that some state‐of‐the‐art systems are beginning to approximate expert‐level performance in tightly defined classification tasks. In the largest multi‐centre study to date [42], a semi‐supervised convolutional neural network achieved an external validation test accuracy of approximately 94% with an AUC of 0.99. This performance, alongside strong concordance with senior pathologists, suggests that CAD may improve consistency and reduce inter‐observer variability in high‐volume settings. However, strong performance on preselected cytological images is not the same as full clinical equivalence, particularly when algorithms are evaluated on constrained datasets rather than routine diagnostic workflows. This consistency may reduce oversight errors and improve reliability, particularly when used as a ‘second reader’ in high‐volume settings.

AI's quantitative precision may be particularly useful for narrow, well‐defined tasks such as neuroendocrine tumour grading. Shaker et al. [40] demonstrated that automated Ki‐67 index calculation on cell block images achieved almost perfect agreement with manual counting (*r* = 0.968, *κ* = 0.86) and correctly reproduced the pathologist's Ki‐67 grade in 92% of cases. The authors concluded that the algorithm performed best as an adjunct rather than a replacement, enhancing objectivity and efficiency while preserving the pathologist's interpretive role. This reflects a broader theme across the literature; CAD appears strongest when deployed to enhance objectivity, standardisation and workflow efficiency within bounded subtasks, while the pathologist retains responsibility for higher‐order interpretation and final diagnosis.

Despite these encouraging results, our review highlights major limitations that temper enthusiasm for clinical deployment. The most important, as reflected by the high risk of bias in our QUADAS‐2 assessment (Figure 2), is that real‐world pancreatic cytopathology is rarely binary. Many included algorithms were trained and tested on retrospectively collected datasets enriched for definitive benign and malignant cases, creating a risk of spectrum bias and potentially inflating performance estimates. In several studies, indeterminate categories were excluded, relabelled or underrepresented, even though atypical and suspicious diagnoses are among the most clinically consequential outputs of pancreatic FNA. This matters because these ‘grey zone’ cases are precisely where human expertise is most valuable. When AI models were challenged with such cases, performance dropped. Fang et al. reported accuracy of 78.79% in atypical cytology, while Momeni‐Boroujeni et al. reported only 77% accuracy in an atypical dataset, compared with perfect performance in definitive benign and malignant cases. These findings suggest that current CAD systems perform best in morphologically clear‐cut cases, but remain less reliable in equivocal lesions where the distinction between benign and malignant cytology is less definite.

A second limitation is that the performance of many models depended on upstream human curation. Most studies did not evaluate autonomous whole‐slide workflows, but instead relied on pathologist‐selected hot spots or representative regions of interest for image capture. In some cases, only diagnostically representative cell clusters were retained. As a result, these systems often analysed enriched fields rather than the full complexity of routine slides, likely inflating performance and limiting generalisability. This was further constrained by small, homogeneous datasets, often from single centres using one stain, preparation method or imaging system. External validation was uncommon, and where performed, performance often declined. For example, Lin et al.'s ROSE‐AI model showed a drop in positive predictive value from 71.7% internally to 60.7% externally [32]. Together, these findings suggest that current CAD systems remain vulnerable to variation in case mix, slide preparation and institutional workflow.

Another limitation is the narrow pathological spectrum represented in current training sets. Most studies focused predominantly on common pancreatic malignancies, particularly PDAC, while rare entities such as acinar cell carcinoma, lymphoma, metastatic lesions or mixed neoplasms were seldom represented or were explicitly excluded. A system trained largely on a benign‐versus‐PDAC binary may perform unpredictably when confronted with rare lesions or unusual mimics, especially when those entities share only partial morphological overlap with the patterns learned during training. Future datasets must therefore expand not only in size but also in pathological breadth if CAD is to support real diagnostic practice rather than idealised binary classification.

This adjunctive role may be particularly valuable in rapid on‐site evaluation (ROSE), where time pressure and workforce limitations are pronounced. Several groups explored AI‐assisted ROSE in EUS‐FNA workflows, a clinically important development because ROSE can improve adequacy and diagnostic yield but is often unavailable outside tertiary centres due to limited on‐site cytopathology support. Lin et al. developed a Diff‐Quik‐based ROSE‐AI model and reported strong external accuracy [32], while Thosani et al. prospectively evaluated ROSE‐AId against in‐person cytopathology [43]. Zhang et al. further combined convolutional and transformer‐based architectures to analyse ROSE images with state‐of‐the‐art performance [37]. Taken together, these studies suggest that AI may have genuine service‐level utility in supporting adequacy assessment and rapid slide triage during procedures. However, even in this setting, the most defensible role is supplementation rather than substitution, particularly when diagnostically equivocal material or uncommon tumour types are encountered.

Despite promising pooled accuracy, clinical translation remains limited by poor handling of indeterminate and non‐diagnostic cases and, more broadly, by poor workflow fit. Future studies should prospectively evaluate CAD within the full EUS‐FNA pathway using unselected whole‐slide datasets, including specimen adequacy assessment, standardised image acquisition and case‐level aggregation without manual ‘hot‐spot’ selection. Reporting should explicitly state how non‐diagnostic specimens and indeterminate categories (atypical/suspicious) are handled, since these drive the most clinically consequential decisions and may depress apparent performance compared with curated binary datasets. Pre‐specifying decision thresholds linked to actions, such as repeat sampling, ancillary testing, MDT discussion or surgical referral, would also improve interpretability and comparability across studies.

## 5 Conclusion

Computer‐assisted methods in pancreatic FNA cytology show considerable promise, with evidence of high diagnostic accuracy. These tools can achieve strong performance in defined classification tasks and offer advantages in speed, consistency and quantitative analysis. AI has demonstrated value as an assistive technology, from supporting ROSE and preliminary diagnosis to objectively grading proliferation markers in PanNETs, with potential to improve workflow efficiency and diagnostic confidence.

Nevertheless, most current models have been developed in controlled settings and frequently simplify diagnosis to binary outcomes, which do not reflect the full complexity of cytopathology. Real‐world deployment will require careful attention to bias, generalisability and clinical integration. Algorithms should be trained on diverse datasets that include atypical, indeterminate and rare lesions, and undergo robust external validation across multiple centres before routine clinical adoption. Future research should prioritise prospective multicentre studies that evaluate AI within real‐time diagnostic workflows and compare AI‐assisted practice directly with standard care. Transparent reporting and adherence to diagnostic accuracy standards will also be essential to support reproducibility and meaningful comparison across studies. Close collaboration between developers, pathologists and initiatives such as the National Pathology Imaging Co‐operative will be important to refine these tools, support implementation and build user trust. With continued validation, AI‐driven image analysis may become a reliable adjunct in pancreatic cytology, improving efficiency and supporting diagnosis under expert human oversight.

## Author Contributions

The original study concept was proposed by Roberto Dina, who also contributed to supervision, methodology, interpretation of results, and critical review and editing of the manuscript. Al‐Amaan Mohamed Mirzan contributed to developing the study design, literature searching, screening, data extraction, formal analysis, visualisation, interpretation of results, and writing of the original draft. Both authors have read and agreed to the version of the manuscript to be published.

## Funding

This research received no specific grant from any funding agency in the public, commercial or not‐for‐profit sectors. The open access article processing charge was paid by the Imperial College Open Access Fund.

## Conflicts of Interest

The authors declare no conflicts of interest.

## Supporting information


**Appendix S1:** cyt70092‐sup‐0001‐AppendixA.docx.

**Table S1:** Reconstructed 2 × 2 contingency data for diagnostic accuracy meta‐analysis.

## Data Availability

The data that supports the findings of this study are available in the Supporting Information of this article.
