# Supplementary material for: What Are Computer‐Assisted Methods Achieving in Fine‐Needle Aspiration Cytology of the Pancreas? A Systematic Review and Meta‐Analysis
Source: Cytopathology. 2026 May 26;37(6):587–602. doi: 10.1111/cyt.70092 (PMC13639511; doi:10.1111/cyt.70092)
Supplement: Supplementary file 1 [file CYT-37-587-s001.docx]

# Appendix A

# Complete Search Strategy for Systematic Review and Meta-Analysis

A systematic literature search was initially conducted using a predefined, focused search strategy (see Section A). However, this initial strategy returned an insufficient number of studies for meta-analysis. Therefore, a broader, second search strategy was developed (see Section B). This document includes both sets of searches in full, allowing reproducibility across databases.

## Section A: Initial Search Strategy (Targeted for PNETs)

Databases: Ovid MEDLINE, Embase, Web of Science, Scopus, IEEE Xplore
Focus: Artificial intelligence in fine needle aspiration (FNA) cytology for pancreatic neuroendocrine tumours (PNETs).
Limits: English language, human studies, publication year from 2010 onward.

### Ovid MEDLINE

1. Artificial Intelligence.mp. or exp Artificial Intelligence/
2. exp Pattern Recognition, Automated/ or Machine Learning.mp. or exp Machine Learning/ or exp Diagnosis, Computer-Assisted/
3. exp Neural Networks, Computer/ or Neural Networks.mp.
4. Computational Pathology.mp. or exp Image Interpretation, Computer-Assisted/
5. Fine Needle Aspiration.mp. or exp Biopsy, Fine-Needle/
6. Cytology.mp. or exp Cytology/
7. Morphology.mp.
8. Cytopathology.mp.
9. 1 or 2 or 3 or 4
10. 5 or 6 or 7 or 8
11. pancreatic neoplasms/ or carcinoma, islet cell/
12. PNET.mp.
13. Pancreatic NET.mp.
14. Neuroendocrine Tumors/ or neuroendocrine tumour.mp. or neuroendocrine tumor.mp.
15. 11 or 12 or 13 or 14
16. 9 and 10 and 15
17. limit 16 to (English language and humans)

### Embase

1. artificial intelligence.mp. or exp artificial intelligence/
2. machine learning.mp. or exp machine learning/
3. deep learning.mp. or exp deep learning/
4. exp artificial neural network/ or neural network*.mp.
5. 1 or 2 or 3 or 4
6. exp aspiration cytology/ or cytology.mp. or exp cytology/
7. exp morphology/ or morphology.mp.
8. cytopathology.mp. or exp cytopathology/`
9. fine needle aspiration.mp. or exp fine needle aspiration biopsy/
10. 6 or 7 or 8 or 9
11. pancreatic neuroendocrine tumor.mp. or exp pancreas islet cell tumor/
12. pancreatic neuroendocrine tumour.mp.
13. PNET.mp.
14. neuroendocrine tumor.mp. or exp neuroendocrine tumor/
15. neuroendocrine tumour.mp.
16. Pancreatic NET.mp.
17. 11 or 12 or 13 or 14 or 15 or 16
18. 5 and 10 and 17
19. limit 18 to (English language and humans)

### Web of Science

TS=("Artificial Intelligence" OR "Machine Learning" OR "Deep Learning" OR "Neural Network*" OR
"Computational Pathology" OR "AI") AND
TS=("Fine Needle Aspiration" OR "FNA" OR "Cytology" OR "Morpholog*" OR "Cytopatholog*") AND
TS=("Pancreatic Neuroendocrine Tumor*" OR "PNET" OR "Pancreatic NET" OR "Islet Cell Tumor*" OR
"Neuroendocrine Tumour" OR "Neuroendocrine Tumor") AND LANGUAGE: (English)

### Scopus

ALL("Artificial Intelligence" OR "Machine Learning" OR "Deep Learning" OR "Neural Network*") AND
ALL("Fine Needle Aspiration" OR "FNA" OR "Cytology" OR "Morpholog*" OR "Cytopatholog*") AND
ALL("Pancreatic Neuroendocrine Tumor*" OR "Pancreatic Neuroendocrine Tumour*" OR "PNET" OR "Pancreatic NET" OR
"Islet Cell Tumor*" OR "Neuroendocrine tumour" OR "Neuroendocrine tumor") AND
LIMIT-TO (LANGUAGE , "English") AND LIMIT-TO (EXACTKEYWORD , "Human")

### IEEE Xplore

(("Artificial Intelligence" OR "Machine Learning" OR "Deep Learning" OR "Neural Network*") AND
("Fine Needle Aspiration" OR "FNA" OR "Cytology" OR "Morpholog*" OR "Cytopatholog*") AND
("Pancreatic Neuroendocrine Tumor*" OR "Pancreatic Neuroendocrine Tumour*" OR "PNET" OR "Pancreatic NET" OR
"Islet Cell Tumor*" OR "Islet Cell Tumour*" OR "Neuroendocrine tumour" OR "Neuroendocrine tumor"))

## Section B: Second Search Strategy (Broader Terms)

Databases: Ovid MEDLINE, Embase
Focus: Artificial intelligence in FNA cytology across all pancreatic lesions, not limited to PNETs.
Limits: English language, human studies, publication year from 2010 onward.

### Ovid MEDLINE

1. Artificial Intelligence.mp. or exp Artificial Intelligence/
2. exp Pattern Recognition, Automated/ or Machine Learning.mp. or exp Machine Learning/ or exp` Diagnosis, Computer-Assisted/
3. exp Neural Networks, Computer/ or Neural Networks.mp.
4. Computational Pathology.mp. or exp Image Interpretation, Computer-Assisted/
5. Digital Image Analysis.mp.
6. 1 or 2 or 3 or 4 or 5
7. Fine Needle Aspiration.mp. or exp Biopsy, Fine-Needle/
8. Cytology.mp. or exp Cytology/
9. Morphology.mp.
10. Cytopathology.mp.
11. 7 or 8 or 9 or 10
12. pancreatic neoplasms/ or carcinoma, islet cell/
13. exp Pancreas/
14. exp Pancreatic Diseases/
15. (pancrea* adj3 (lesion* or mass* or cancer* or carcinoma* or tumo?r* or neoplas* or malignan*)).mp.
16. 12 or 13 or 14 or 15
17. 6 and 11 and 16
18. limit 17 to yr="2010"

### Embase

1. artificial intelligence.mp. or exp artificial intelligence/
2. machine learning.mp. or exp machine learning/
3. deep learning.mp. or exp deep learning/
4. exp artificial neural network/ or neural network*.mp.
5. exp computer assisted diagnosis/
6. exp image analysis/ or exp image interpretation, computer assisted/ or digital image analysis.mp.
7. exp aspiration cytology/ or cytology.mp. or exp cytology/
8. exp morphology/ or morphology.mp.
9. cytopathology.mp. or exp cytopathology/
10. fine needle aspiration.mp. or exp fine needle aspiration biopsy/
11. 5 or 6 or 7 or 8
12. exp pancreas tumor/
13. exp pancreas cancer/
14. (pancrea* adj3 (lesion* or mass* or cancer* or carcinoma* or tumo?r* or neoplas* or malignan*)).mp.
15. 12 or 13 or 14
16. 1 or 2 or 3 or 4 or 14 or 15
17. 9 and 15 and 16
18. limit 17 to yr="2010"

### Web of Science

TS=("Artificial Intelligence" OR "Machine Learning" OR "Deep Learning" OR "Neural Network*" OR "Computational Pathology" OR "Computer Aided Diagnosis" OR "Computer Assisted Diagnosis" OR "Digital Image Analysis")

AND

TS=("Fine Needle Aspiration" OR "FNA" OR "Fine Needle Biopsy" OR "FNB" OR "Cytology" OR "Morpholog*" OR "Cytopatholog*")

AND

TS=("Pancreas" OR "Pancreatic Lesion*" OR "Pancreatic Mass*" OR "Pancreatic Neoplasm*" OR "Pancreatic Cancer*" OR "Pancreatic Carcinoma*" OR "Pancreatic Adenocarcinoma" OR "Pancreatic Tumo?r*")

### Scopus

TITLE-ABS-KEY ( "Artificial Intelligence" OR "Machine Learning" OR "Deep Learning" OR "Neural Network*" OR "Computational Pathology" OR "Computer Aided Diagnosis" OR "Computer Assisted Diagnosis" OR "Digital Image Analysis" )

AND TITLE-ABS-KEY ( "Fine Needle Aspiration" OR "FNA" OR "Fine Needle Biopsy" OR "FNB" OR "Cytology" OR "Morpholog*" OR "Cytopatholog*" )

AND TITLE-ABS-KEY ( "Pancreas" OR "Pancreatic Lesion*" OR "Pancreatic Mass*" OR "Pancreatic Neoplasm*" OR "Pancreatic Cancer*" OR "Pancreatic Carcinoma*" OR "Pancreatic Adenocarcinoma" OR "Pancreatic Tumo?r*" ) A

ND PUBYEAR > 2009 AND PUBYEAR < 2026

AND ( LIMIT-TO ( LANGUAGE , "English" ) )

### IEEE Xplore

(("Artificial Intelligence" OR "Machine Learning" OR "Deep Learning" OR "Neural Network*" OR "Computational Pathology" OR "Computer Aided Diagnosis" OR "Computer Assisted Diagnosis" OR "Digital Image Analysis") AND ("Fine Needle Aspiration" OR "FNA" OR "Fine Needle Biopsy" OR "FNB" OR "Cytology" OR "Morpholog*" OR "Cytopatholog*") AND ("Pancreas" OR "Pancreatic Lesion*" OR "Pancreatic Mass*" OR "Pancreatic Neoplasm*" OR "Pancreatic Cancer*" OR "Pancreatic Carcinoma*" OR "Pancreatic Adenocarcinoma" OR "Pancreatic Tumor*" OR "Pancreatic Tumour*"))
