# Supplementary material for: What Are Computer‐Assisted Methods Achieving in Fine‐Needle Aspiration Cytology of the Pancreas? A Systematic Review and Meta‐Analysis
Source: Cytopathology. 2026 May 26;37(6):587–602. doi: 10.1111/cyt.70092 (PMC13639511; doi:10.1111/cyt.70092)
Supplement: Supplementary file 2 [file CYT-37-587-s002.docx]

| Author | Dataset used | Analysis Level | | N_Pos | | N_Neg | TP | FP | TN | FN |
| --- | --- | --- | --- | --- | --- | --- | --- | --- | --- | --- |
| Fang 2025 | SSCNN External Testing | Image | 109 | | 22 | | 102 | 1 | 21 | 7 |
| Fuji 2024 | Geometric Transformation | Image | 2079 | | 2080 | | 1900 | 312 | 1768 | 179 |
| Lin 2023 | ROSE-AI External Test | Image | 109 | | 584 | | 85 | 55 | 529 | 24 |
| Qin 2023 | ResNet18-HSI-SimSiam Add'l Test Set | Image | 101 | | 93 | | 95 | 9 | 84 | 6 |
| Zhang 2023 | PUMCH cytology images | Image | 304 | | 544 | | 284 | 16 | 528 | 20 |
| Kong 2019 | Digital Image Analysis (DIA) | Patient | 102 | | 40 | | 86 | 8 | 32 | 16 |
| Momeni-Boroujeni 2017 | MNN Testing dataset | Patient | 22 | | 35 | | 22 | 0 | 35 | 0 |
| Zhang 2022 | DCNN Pooled External Testing | Patient | 46 | | 39 | | 45 | 1 | 35 | 4 |
| Khozeymeh 2023 | DIA - Sum of 3 Factors (N=150 Test) | Sample | 90 | | 60 | | 55 | 8 | 52 | 35 |
| Yamada 2022 | MTC Multivariate (D=50, specific indices) | Specimen | 143 | | 103 | | 111 | 31 | 72 | 32 |

**Supplementary Table 1: Reconstructed 2×2 Contingency Data for Diagnostic Accuracy Meta‐Analysis**

This table presents the reconstructed 2×2 contingency data for each study included in our meta‐analysis. For each paper, we specify the dataset and analysis level (images, patients, samples, or specimens), the total numbers of disease‐positive (N_Pos) and disease‐negative (N_Neg) cases, and the derived true positives (TP), false negatives (FN), true negatives (TN), and false positives (FP). These cell counts were computed by multiplying N_Pos by the reported sensitivity (to obtain TP, with FN = N_Pos – TP) and N_Neg by the reported specificity (to obtain TN, with FP = N_Neg – TN), then rounding to the nearest whole case. For Fujii et al. 2024 (geometric transformation), counts are aggregated over all five cross-validation folds, and for Zhang et al. 2022 (DCNN pooled external testing) they are summed across the three external hospitals. The Yamada et al. 2022 “MTC Multivariate (D = 50)” entry refers to the 50-feature model applied to the full 246-specimen cohort.
